# Prevalence rate of undiagnosed tuberculosis in the community in Ethiopia from 2001 to 2014: systematic review and meta-analysis

**DOI:** 10.1186/s13690-019-0360-2

**Published:** 2019-07-11

**Authors:** Balew Arega, Kelemu Tilahun, Abraham Minda, Asnake Agunie, Getachew Mengistu

**Affiliations:** 1 Yekatit 12 Hospital Medical College, Addis Ababa, Ethiopia; 2 College of Health Science, Wolega University, Wolega, Ethiopia; 3 College of health science, Debere Markos University, Debere Markos, Ethiopia

**Keywords:** Undiagnosed PTB, Smear positive, Prevalence, Community, Ethiopia

## Abstract

**Background:**

In Ethiopia individual report indicated nearly 30% of incident cases of tuberculosis remained undiagnosed. Therefore, this systematic review and meta-analysis was aimed to determine the pooled prevalence rate of undiagnosed smear positive pulmonary tuberculosis (PTB) using community based studies published in Ethiopia.

**Methods:**

MEDLINE/PubMed, ’Cochrane’ library, and Google scholar databases were searched, and reference list of studies on tuberculosis in Ethiopia were reviewed. We used table to present descriptive information of original studies and quantitative results were presented in forest plots. The Cochrane Q test and I^2^ test statistic were used to test heterogeneity across studies. The Pooled prevalence and point estimates of undiagnosed smear positive PTB were computed by a random effects model.

**Results:**

From the nine studies included in the analysis, the pooled prevalence rate and point estimate of undiagnosed smear positive PTB was 0.11%(95% CI, 0.06–013%, *p* < 0.001) and 79.8/100,000(95% CI; 56.3–112.8) respectively. Pooled prevalence rate and point estimate of bacteriologically confirmed PTB were 0.17%(95%CI; 0.13–0.22%, *P* < 0.001) and 191/100000(95% CI; 141.3–258) respectively. The ratio of active to passive case detection was 2.3(95% CI, 0.42–4.1). Pooled prevalence rate of presumptive PTB was 2.7%(95% CI; 1.3–5.3%).

**Conclusions:**

The analysis revealed that the magnitude of undiagnosed smear positive PTB cases in the community is high in Ethiopia. This indicated the ongoing transmission of tuberculosis in community due to missed infectious cases. Active tuberculosis finding in the community should be strengthened in Ethiopia.

**Trial registration:**

140611.

## Background

Tuberculosis (TB) is a chronic infectious disease caused by *Mycobacterium tuberculosis* (MTB) and mainly affects lungs (PTB) [1]. Among many factors influencing transmission, smear positivity and Age (being adult) are strongly predicts which patients are the most contagious [2]. About 80% of tuberculosis transmission was due to smear positive pulmonary tuberculosis [3].

Despite the progress made in the last two decades and saved more than five million lives, TB remains one of the world’s most devastating infectious diseases [4]. A major problem hampering control efforts and driving the TB epidemic is the large reservoir of undiagnosed smear positive PTB disease which comprise ~ 30 to 50% of the total TB burden [5]. Every year, one in three people who fall ill with TB are left undiagnosed or not registered by health systems mainly in TB high burden countries [6].

The major cause of undiagnosed cases of tuberculosis in TB high burden countries including Ethiopia could be related the following reasons. The first is the use of passive case detection (PCD) strategy that is deepened on self-presentation of symptomatic patients to the health system for diagnosis instead of active cases detection (ACD) which identify and bring people with TB who not sought diagnostic services on their own initiative [5, 7]. The second reason could be use of low sensitivity diagnostic technique such as Ziehl Neelsen (ZN) microcopy, remains the cornerstone of diagnosis modalities in low income countries [8], rather than using a relatively sensitive test such as fluoresce microscopy and highly sensitive tests including molecular technique for a couple of reasons [6].

Ethiopia achieved the millennium goal on TB by halving the incidence of tuberculosis in 2015 [9]. On the other hand, the country is still among the 22 high TB burden countries [10]. This might be related to a high burden of undiagnosed or missed (30% of incident cases) as showed with individual reports in the country [11]. Better understanding of undiagnosed smear positive PTB is an essential epidemiological index to estimate the burden in a community and has important implications for prevention of transmission [12]. Therefore, this study aimed to answer the questions:- 1) What is the magnitude of undiagnosed smear positive pulmonary tuberculosis in community in Ethiopia among potentially infectious age groups?, 2) what is the weighted mean ratio of active to passively detected pulmonary tuberculosis cases in the included studies?. For this purpose, cross sectional studies conducted in Ethiopia were systematically reviewed and combined.

## Methods

### Study design and search strategy

This review sets using published studies to estimate the prevalence of undiagnosed smear positive PTB in community in Ethiopia. Studies were searched using *MEDLINE/PubMed*, ‘Cochrane’ library and Google scholar databases as well as reviewing reference list of previous studies on Tuberculosis in Ethiopia. The search was done following the Preferred Reporting Items for Systematic Reviews and Meta-Analyses (PRISMA) statement guideline [13]. The electronic search was performed for the MEDLINE/PubMed database using the following Medical Science Heading (MeSH) terms: (“tuberculosis”[MeSH Terms] OR “tuberculosis”[All Fields]) OR TB[All Fields] OR MTB [All Fields] OR (“*Mycobacterium tuberculosis*”[MeSH Terms] OR (“mycobacterium”[All Fields] AND “tuberculosis”[All Fields]) OR “*Mycobacterium tuberculosis*”[All Fields]) AND (“community”[MeSH Terms] OR “community”[All Fields]) OR (“population”[MeSH Terms] OR “population”[All Fields]) OR (“survey”[MeSH Terms] OR “survey”[All Fields] OR (“screening”[MeSH Terms] OR screening [All Fields]) AND (“Ethiopia”[MeSH Terms] OR “Ethiopia”[All Fields]).The study limited only English language studies and human being category.

### Study selection and inclusion criteria

Studies were selected for this review if conducted in Ethiopia. The studies were assessed by two independent researchers against the following inclusion criteria:-1) Community based cross sectional study done in the community or population; 2) The age groups > 14 years; 3) reported type of laboratory tests performed; 4) Study using sputum sample; 5) Pulmonary tuberculosis;7) studies that reported quality assurance methods. Other studies reported the knowledge and practice of individuals towards TB disease and investigated patterns of drug resistance only were excluded. It was also not included reviews that reiterated findings from the already included studies not to repeat used the same data in a single review which is not meaningful.

### Data extraction/abstraction

The data extraction was done by two researchers (BA, GM) using a standardized and pretested format. Data were extracted using Microsoft Excel and includes: title, first author, publication year, year of survey, design of the study, regions of study (study site in the country),study base (population-based or Community based), sample size, number of suspected case, type of laboratory test (Culture or Acid Fast Bacilli (AFB) Microscopy), age group of study participants, number of newly diagnosed smear positive PTB, number of bacteriologically confirmed (old Vs. new) tuberculosis, actively Vs. passively diagnosed (on treatment). The point prevalence rate of undiagnosed smear positive and total bacteriological confirmed point prevalence of PTB was determined for each study. Disagreements on data extractions between the two investigators were solved by discussion and consensus.

### Quality assessment

The quality of the included studies was evaluated by two reviewers using Joanna Briggs Institute (JBI) appraisal tool for prevalence studies [14]. These assessment criteria include 11 criteria with three potential responses: yes, no and not mentioned. The criteria are**:** − 1) was the sample frame appropriate to address the target population? 2) Were study participants sampled in an appropriate way? 3) Was the sample size adequate? 4) Were the study subjects and the setting described in detail? 5) Was the data analysis conducted with sufficient coverage of the identified sample? 6) Were valid methods used for the identification of the condition? 7) Was the condition measured in a standard, reliable way for all participants? 8) Was there appropriate statistical analysis? 9) Was the response rate adequate, and if not, was the low response rate managed appropriately All assessments were entered into pre-performed and standardized data extraction forms. Studies were evaluated for quality by using these indicators; those with medium (fulfilling 50% of quality assessment criteria) and high quality were included for analysis. No study was excluded because of poor quality rather excluded due to one or more of the exclusion criteria described above.

### Data analysis

The extracted data were entered and analyzed using Compressive Meta-analysis version 2.2 software. The original articles were described using forest plot and table. Since there was heterogeneity among studies, random effect model was used to compute the pooled prevalence rate of undiagnosed smear positive and total bacteriological confirmed PTB. Random effect model is more conservative than fixed effect model and takes into account any heterogeneity inherent in the meta-analysis. The estimated pooled prevalence rate with its 95% confidence interval (CI) was presented.

### Heterogeneity and publication bias

A potential source of heterogeneity across studies was evaluated by Cochrane Q test (presence of heterogeneity) and I^2^statistics (amount of heterogeneity). The existence of heterogeneity was verified using Cochrane Q test (*P* < 0.10 indicates statistically significant heterogeneity) and I^2^ test that measures level of statistical heterogeneity between studies (values of 25, 50 and 75% are to mean low, medium and high heterogeneity respectively). The Egger weighted regression and Begg rank correlation test methods were used to statistically assess publication bias (*P* < 0.05 was consider as suggestive of statistically significant publication bias).

## Results

### Characteristics of included studies

**Fig. 1:**
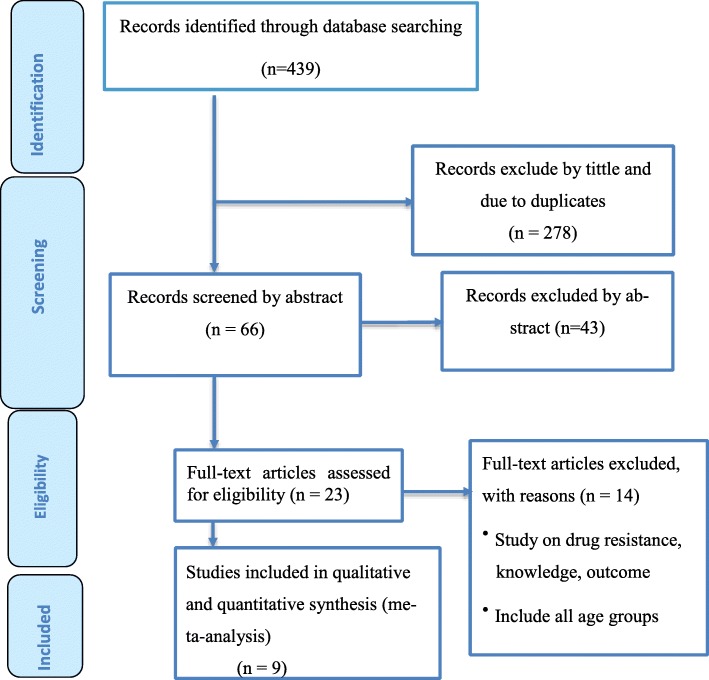
PRISMA flow chart of study selection

The search strategy retrieved 439 potential articles, of which 23 were screened as full text articles and nine studies comprising of 237,648 individuals were found eligible and included in the analysis (Fig. 1). All were community based cross-sectional studies and including PTB cases with a study population ranging from 12,149 individuals in Addis Ababa [15] to 47,478 in Amhara region [16] and conducted from 2001 to 2014.

The studies were conducted in different regions of the country; Southern Nations, Nationalities and Peoples (SNNP) [17], Addis Ababa [15], Amhara [16, 18], Oromia [19, 20], Afar [21] and Tigray region [22]. One study was conducted across regions [23]. All studies used questionnaires for screening of symptoms of PTB followed by diagnostic testing of presumptive PTB patients [15–22]. One study reported chest X-ray as additional screening tool [23]. Among the articles included in the analysis, four studies [15–18] were use Microscopy (ZN or Florescence) for bacteriological confirmation of PTB and the rest five studies combined Microscopy with Culture [19–23]. Three studies provide the information on median duration of cough ranging 13 week [19] to 52 weeks [21]. Only two studies assessed the TB-HIV co-infection one with 1.9% [22] and another with 5.5% [19]. Study characteristics are summarized in Table 1.

**Table 1:** Summary of studies assessing the prevalence undiagnosed smear positive pulmonary tuberculosis in Ethiopia

Study	Region	Study design	Study period	Inclusion Criteria	Diagnostic Method	Sample Size	Presumptive PTB, no (%)	PTB no (%)	New SPPTB,no (%)
Demissie et al 2002 [15]	Addis Ababa	Cross -sectional	May 2001	cough> 2 Week Age ≥ 14 Year	ZN Microscopy	12,149	173(1.4)	23(0.20)	21(0.20)
Shargie et al *2006* [17]	SNNRS	Cross-sectional	February 2003	Cough> 2 Week, Age ≥ 14 Year	ZN Microscopy	16,697	436(2.6)	37(0.22)	13(0.10)
Yimer et al 2009 [16]	Amhara	Cross-sectional	March 2008	cough> 2 Week Age ≥ 15 Year	ZN Microscopy	47,478	1006(2.1)	53(0.12)	38 (0.08)
Tadesse et al *2011* [18]	Amhara	Cross -sectional	Oct- Dec 2010	Cough ≥ 2 Week Age ≥ 14 Year	ZN Microscopy	23,590	831(3.5)	41(0.20))	19(0.08)
Deribew *etal 2012* [19]	Oromia	Cross -sectional	Feb-Mar 2009	Cough ≥ 2 Week Age ≥ 15 Year	ZN Microscopy Culture	27,597	428(1.6)	21(0.10)	5(0.08)
Legessea et al *2013* [21]	Afar	Cross-sectional	Mar-Apr 2010	cough ≥ 2 Week Age ≥ 15 Year	ZN Microscopy Culture	18,192	222(1.2)	62(0.34)	3(0.02)
Berhe et al *2013* [22]	Tigray	Cross-sectional	Mar –Aug 2011	Cough ≥ 2 Week Age ≥ 15 Year	ZN Microscopy Culture	12,175	350(2.9)	30(0.24)	16 (0.13)
Kebede et al *2014* [23]	Various regions	Cross-sectional	Oct 2010 –June 2011	Cough ≥ 2 Week, Age > 15 Year	ZN Microscopy Culture	46,697	5868(12.6	110(0.23)	97 (0.21)
Hamusse et al *2017* [20]	Oromia	Cross-sectional	July 2013–June 2014.	Cough> 2 Week Age ≥ 15 Year	ZN Microscopy Culture	33,073	1041(3.1)	43(0.13)	33(0.10)

**Legends**: ***SNNP*** Southern Nations, Nationalities and Peoples, ***TB*** Tuberculosis, ***SPPTB*** Smear Positive Pulmonary Tuberculosis, ***PTB*** Pulmonary Tuberculosis, ***ZN*** Ziehl-Neelsen, ***LED*** Light Emitting Diode

### Heterogeneity and publication bias

**Fig. 2:**
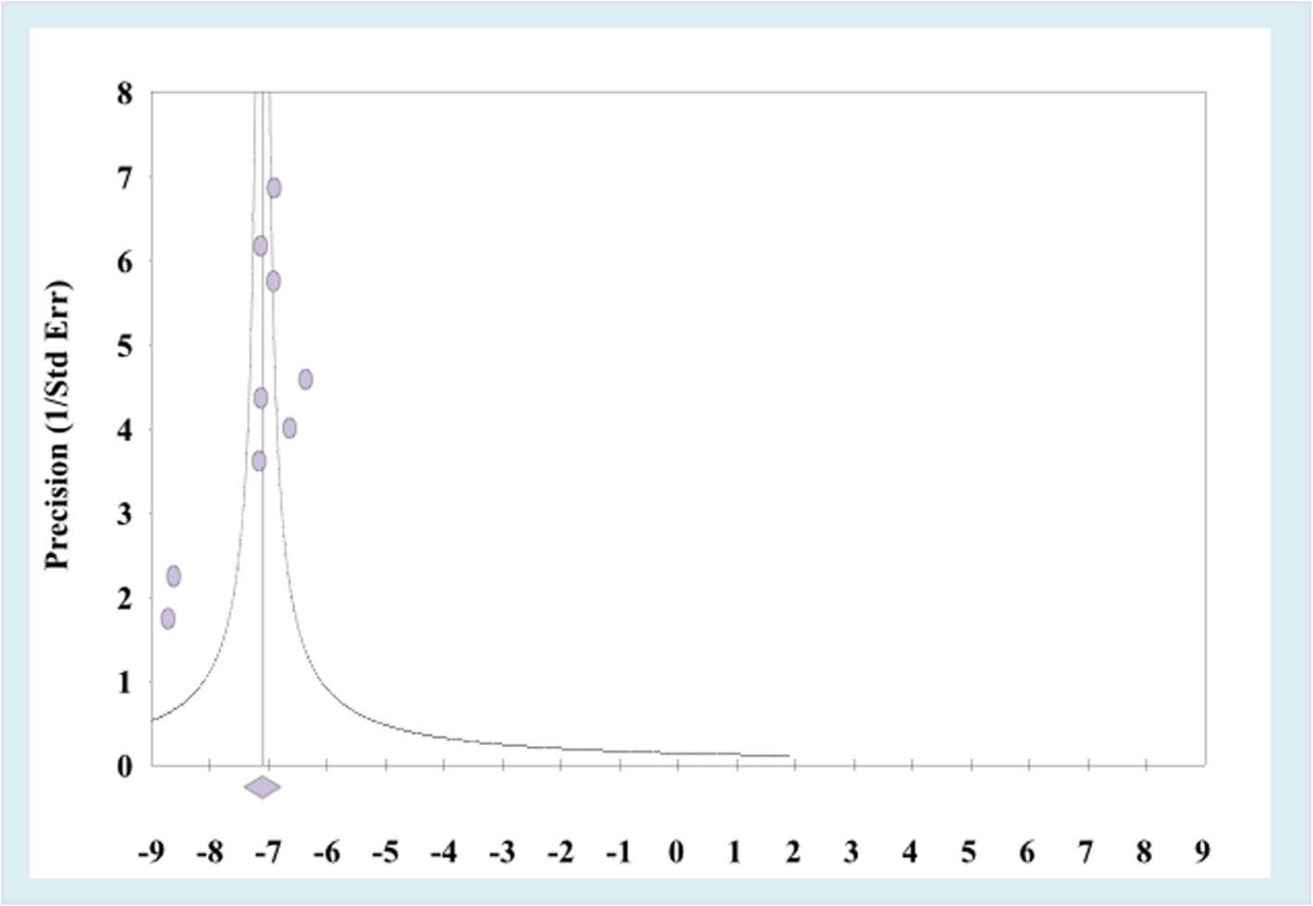
Funnel plot analysis of publication bias

The included articles exhibited high heterogeneity according to Cochrane Q test (Q test *p* = 0.001) and I^2^ test (I^2^ = 88.3%), which is indicative to using random effects model. The Egger weighted regression statistics (*p* = 0.08) and Begg rank correlation statistics (*p* = 0.2) indicated no evidence of publication bias (Fig. 2). As the numbers of the eligible studies included were limited, subgroup analysis to explore the source of heterogeneity is not visible in this systemic review.

### Tuberculosis in the community

**Fig. 3:**
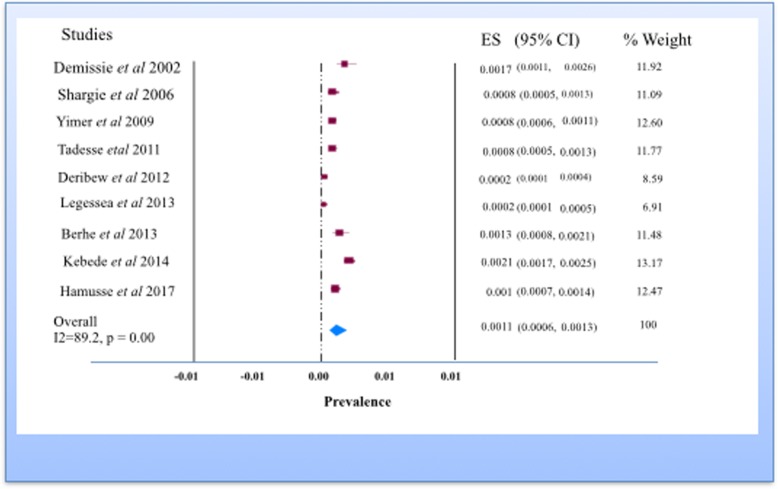
Forest plot of the prevalence rate of undiagnosed smear positive tuberculosis in Ethiopia

The pooled prevalence rate of smear positive undiagnosed PTB (active smear positive PTB detection) from the random effects method of all studies included was 0.11% (95% CI, 0.06–013%, *p* < 0.001) (Fig. 3). The point prevalence estimate (per 100,000) of undiagnosed smear positive PTB in all the studies included was 79.7 (95% CI; 56.3–112.8).

The pooled prevalence rate of bacteriologically confirmed PTB revealed a prevalence of 0.17% (95% CI; 0.13–0.22%, *P* < 0.001). The point prevalence estimate (per 100, 000) of bacteriologically confirmed PTB in all the studies included was 191 (95% CI; 141.3–258).

The weighted mean ratio of actively to passively diagnosed bacteriologically confirmed PTB cases was 2.3 (95% CI, 0.42–4.1). This shows for every one PTB cases detected by the health facility, more than two bacteriologically confirmed PTB cases left undiagnosed in the community. Using cough ≥ 2 weeks as main criterion, the pooled prevalence rate of presumptive PTB in the community was 2.7% (95% CI; 1.3–5.3%, *P* < 0.001).

## Discussion

This systematic review and meta-analysis was the first in type in Ethiopia conducted to estimate the prevalence rates of undiagnosed smear positive PTB among potentially infectious age groups using published studies conducted in Ethiopia. The included studies used various inclusion criteria, diagnostic assays and sampled only presumptive PTB cases. All of the studies reported new (actively detected) and old (on treatment or known) TB patients separately.

The pooled prevalence rate of undiagnosed smear positive PTB in the community in Ethiopia was 0.11%(95% CI, 0.08–0.12%, *p* < 0.001). This is comparable with a study in central India (0.13%) [24] and Vietnam (0.15%) [25]. However, it is lower than a report from Uganda (4.1%) [26] and Nigeria (3.5%) [27]. This difference might be due to the type of microscopy tests used to diagnose PTB while all studies included in this review except one (used florescence) used ZN microscopy. The prevalence of PTB is underestimated by 37% if only symptoms without CXR, which is true in all studies included in this review, are used to identify PTB [28].

The most common source of infection for PTB is smear positive cases in adult age groups [2]. The minimum age in all studies included in this review was 14 years; therefore our result suggested that the missed cases are infectious. In support of this, the first Ethiopia national tuberculosis surveillance revealed that 55% of undetected cases in the community were among the younger age groups (15–34 years) [23].

The pooled prevalence estimate of undiagnosed smear positive PTB in this review (79.8/100,000(95% CI, 56.3–112.8) was slightly lower than study from Korea (93/100,000) [29] and Bangladesh (95/100000) [30]. More than a twofold higher than our result were also found in South India (169/100000) [31] and Philippines (310/100,000) [32].

The overall bacteriologically confirmed pooled prevalence estimate was found to be 191/100,000(95% CI, 141.3–258). This finding was slightly higher than the recent (2017) WHO (164/100,000) estimate of Ethiopia [33], though it was about one-fifth of the point prevalence rate of tuberculosis in the Ethiopian prisons (888/100,000) [34]. This review also noted that nearly half the bacteriologically confirmed PTB cases in the general population were smear positive and undiagnosed.

Further comparison of the epidemiological backgrounds of PTB cases detected by active and passive case finding revealed more than twofold of bacteriologically confirmed PTB cases were left undiagnosed in the community. Similarly, a study in Uganda showed more PTB cases were identified by active case detection [26]. In contrast, a study in South Africa revealed more than four folds of the PTB cases were detected by the health facilities [35]. This may be related to health system in South Africa which is easily accessible and has a strong TB referral system.

According to WHO, individuals who suffered from cough with a duration of more than 2 weeks are expected to visit health institutes for tuberculosis diagnosis [36]. In the current review, however, about 2.7% of individual who had cough for more than 2 weeks was not visited the nearby health facilities. This is equivalent to a study in rural community in the country (2%) [37].

This study had certain limitations. First, it included only published peer-reviewed studies and important data might be missed from unpublished studies. Second, prevalence as a measure of disease burden has limitations as it provides an estimate at a single point in time and cannot distinguish between disease as a result of recent infection and disease from reactivation, limiting understanding of current transmission. Third, lack of information and data from some pastoral region including the Gambella, Somalia and Benshangul-Gumz made it difficult to generalize the findings. Lastly, heterogeneity was relatively high in all analyses. To address the issue of potential variability across studies, the analysis was performed by using random effect model that takes into considerations of any heterogeneity inherent in the meta-analysis and tends to give more conservative estimate. Nevertheless, the main strength of this review was that most of the included studies had large sample sizes. Two investigators independently extracted dataand reviewed the articles to obtain data accurately. We report the results in accordance with the PRISMA statement.

## Conclusion

The analysis revealed a high prevalence of undiagnosed smear positive PTB among potentially infectious age groups in Ethiopia. This could be the potential source for the ongoing transmission of tuberculosis in the community. Therefore, we recommend strengthening of active tuberculosis detection in the country.

## Data Availability

The datasets used and/or analyzed during the current study are available freely upon request of the primary author.

## Abbreviations

AFB: Acid Fast Bacilli
CXR: Chest X-ray
HIV: Human Immunodeficiency Virus
PTB: Pulmonary Tuberculosis
TB: Tuberculosis
